# Low sensitivity of a-defensin (Synovasure) test for intra­operative exclusion of prosthetic joint infection

**DOI:** 10.1080/17453674.2018.1444301

**Published:** 2018-03-06

**Authors:** Ruben Scholten, Jetze Visser, Job L C Van Susante, Corné J M Van Loon

**Affiliations:** Department of Orthopedics, Rijnstate Hospital, Arnhem, The Netherlands

## Abstract

**Background and purpose:**

The Synovasure lateral flow test was developed as a rapid test for the detection or exclusion of periprosthetic joint infection (PJI). 3 studies have reported promising results on its diagnostic value in total joint revision surgery. We aimed to assess the sensitivity and specificity of the Synovasure test to exclude infection in patients undergoing revision surgery for suspected early aseptic loosening of a total hip or knee arthroplasty.

**Patients and methods:**

In a prospective study design, 37 patients who underwent revision surgery for suspected early aseptic loosening (< 3 years after primary arthroplasty) were included. The Synovasure test was used intraoperatively to confirm the aseptic nature of the loosening and 6 tissue cultures were obtained in all cases. Exclusion criteria were patients with a preoperatively confirmed PJI, acute revisions (< 90 days after primary arthroplasty) and cases with malpositioning, wear, or instability of the prosthesis.

**Results:**

5 of the 37 patients were diagnosed with a PJI based on the intraoperative tissue cultures. In only 1 out of these 5 cases this was confirmed by the intraoperative Synovasure test. No tests were falsely positive.

**Interpretation:**

In this case series the Synovasure lateral flow test had a low sensitivity to exclude PJI in patients with suspected aseptic loosening. The role of the Synovasure lateral flow test in the intraoperative exclusion of PJI during revision surgery for suspected early aseptic loosening appears to be more limited than previously indicated.

Periprosthetic joint infection (PJI) accounts for up to 25% of failed total knee arthroplasties (TKA) and up to 15% of failed total hip arthroplasties (THA) (Bozic et al. 2009, 2010).

Distinguishing the septic from the aseptic failures in total joint arthroplasty (TJA) is critical in the successful treatment of painful prosthetic joints, as they require different surgical strategies. To this end, the Musculoskeletal Infection Society has formulated criteria for decision-making in the work-up of suspected of PJI (Parvizi et al. 2011). However, this work-up remains far from straightforward, mainly since there is no uniform test for diagnosing PJI (Parvizi et al. 2011). Therefore, a simple diagnostic tool for PJI would be important.

Recently, the presence of the α-defensin biomarker in synovial fluid was suggested as a possible marker of periprosthetic joint infection, since it is naturally released by neutrophils in the presence of synovial fluid pathogens (Ganz et al. 1985, Deirmengian et al. 2015b). 3 studies using quantitative measurements of α-defensin have reported a sensitivity and specificity above 96% for PJI (Deirmengian et al. 2014a, 2014b, 2015a).

From these reported high sensitivity and specificity of α-defensin levels in detecting PJI, the Synovasure test (Zimmer Inc., Warsaw, IN, USA), a lateral flow test for the detection of α-defensin levels, has been made commercially available. The Synovasure lateral flow test is a rapid measure of the α-defensin levels in synovial fluid, and provides dichotomic results after 10 minutes. 2 studies reported a sensitivity (67–69%) and specificity (93–94%) lower than in the previously mentioned quantitative measurements (Kasparek et al. 2016, Sigmund et al. 2017). However, a recent study by Berger et al. (2017) reported higher sensitivity (97%) and specificity (97%).

The previously mentioned studies were performed in non-specific patient populations containing varying indications for revision surgery ranging from evident acute PJI to reimplantation in 2-stage PJI revisions. However, the true clinical value of this promising test may lie in its ability to intraoperatively distinguish the early (< 3 years following implantation) aseptic failure, where a 1-stage revision is indicated, from the early septic failure with less virulent micro-organisms where a staged revision would be more appropriate.

In this study we evaluated the additional value of the intraoperative Synovasure lateral flow test in confirming the absence of PJI in a group of patients undergoing prosthetic joint revision surgery for suspected early aseptic loosening. The results of the Synovasure test were compared with intraoperative tissue cultures. Furthermore, we assessed the possible correlation of false-negative test results with the presence of metallosis, since previous studies have suggested that metallosis may predispose to false-positive results (Bonanzinga et al. 2017). 

## Patients and methods

Since August 2015, the Synovasure test has been used in our clinic as an adjunct tool to exclude PJI intraoperatively in revision patients with suspected early aseptic loosening of an implanted THA or TKA. Cases were prospectively included in the presence of a chronically painful (> 90 days) prosthetic joint in those who underwent revision surgery due to suspected early aseptic loosening (< 3 years after primary arthroplasty) of the implant after TKA or THA between August 2015 and October 2017. During revision surgery on these patients in this period, the Synovasure test was used to aid in the exclusion of PJI. Excluded from this study were patients already diagnosed with PJI according to the MSIS criteria, acute revisions (< 90 days), revisions due to dislocations, revisions due to malpositioning, or cases where an insufficient amount of synovial fluid could be aspirated to perform the Synovasure lateral flow test.

In all cases, synovial fluid was aspirated under aseptic conditions (after surgical dissection up to the joint capsule) from the affected joint whilst avoiding any contamination with blood. The Synovasure test was carried out according to the manufacturer’s instructions. In the case of a positive Synovasure test, we considered the joint to be infected and proceeded with the removal of the prosthesis and implantation of a spacer containing antibiotics. If the test was negative, we proceeded with a one-stage revision of the affected joint. In all cases, a total of 6 microbiologic cultures of synovial tissue and the interface membrane were collected. The tissue samples were cultured for 14 days in the microbiology laboratory. Tissue cultures were considered positive for PJI when at least 2 out of 6 cultures grew identical pathogens. During surgery, the presence of metallosis or macroscopic signs of infection (presence of pus) were noted.  

### Ethics, funding, and potential conflicts of interest

The procedures followed were in accordance with the ethical standards of the responsible committee on human experimentation and with the Helsinki Declaration of 1975, as revised in 2000. This research received no specific grant from any funding agency in the public, commercial, or not-for-profit sectors. No competing interests were declared.

## Results

37 patients (22 men), mean age 66 (51–81) years, planned for revision surgery met the inclusion criteria. Revision surgery was performed on 8 hips and 29 knees. 5 patients were diagnosed with PJI due to positive tissue cultures. Of these, 1 patient had a positive Synovasure test and 4 patients tested negative. Thus, there were 1 true-positive Synovasure test, and 4 false-negative Synovasure tests (Table). No false-positive Synovasure tests were observed, even in the presence of metallosis.

**Table.** Specification of false-negative cases

False-negative case no.	Identified pathogens	Positive cultures
1	*Staphylococcus epidermidis*	2/6
2	*Staphylococcus epidermidis*	3/6
3	*Staphylococcus epidermidis* and	
	*Staphylococcus saccharolyticus*	2/6
4	*Propionibacterium acnes*	2/6

4 false-negative cases were observed; all these patients were treated as 1-stage revisions for PJI with adequate antibiotics over the course of 12 weeks.

The only true positive test occurred in a patient with intraoperative macroscopic signs of infection due to the presence of pus. In this patient, previous tests did not indicate PJI according to the MSIS criteria; however, 6 intraoperative tissue cultures grew *Staphylococcus epidermidis*. 29 true negative tests were observed; in 1 of these cases metallosis was present during surgery.

## Discussion

The identified sensitivity (1/5) is clearly lower than reported in previous studies (Kasparek et al. 2016, Berger et al. 2017, Sigmund et al. 2017). These 3 earlier studies reported a sensitivity of 67–97% and specificity of 93–96% of the Synovasure test for the diagnosis of PJI

In contrast to our study these studies included various kinds of procedures, namely: patients fulfilling the MSIS criteria for PJI preoperatively, 1-stage revisions, reimplantations at second-stage revision, explantations and spacer implantations, spacer exchanges, debridements with exchange of mobile parts and retention of the prosthesis and excision of a hip prosthesis (Sigmund et al. 2017). Kasparek et al. (2016) also included cases with varying indications: aseptic loosening, polyethylene wear with osteolysis, suspected chronic PJI, patients fulfilling the MSIS criteria for PJI preoperatively, instability and stiffness. Berger et al. (2017) included patients fulfilling a modified version of the MSIS criteria for PJI preoperatively. This modification, and the non-specific patient populations including varying indications for revision surgery, makes the reported values difficult to interpret and compare. Even more so in establishing the tests’ ability to distinguish early aseptic failure from septic failure in unclear cases that are not evidently infected (not fulfilling the modified MSIS criteria for PJI).

In contrast to these earlier studies our study focused on the ability of the Synovasure test to exclude PJI in a uniform subgroup of patients undergoing revision surgery for suspected early aseptic loosening. To our knowledge, this is the first study to assess the Synovasure lateral flow test in this specific homogeneous subgroup of patients. From clinical practice this is an important strength of our study and as such is the finding of a rather low sensitivity in this particular subgroup. This strength has to be balanced against the limitation of a rather small number of patients included, which warrants caution in drawing firm conclusions. Another strength of our study, and that of Sigmund et al. (2017), is that there is no conflict of interest in relation to the manufacturer of the Synovasure test.

Previous studies have suggested that the presence of metallosis may predispose to false-positive results of the Synovasure test. In our study, there was 1 case of metallosis, which yielded a negative Synovasure test.

Our findings indicate that the Synovasure lateral flow test has limited additional value for the intraoperative exclusion of PJI from low virulent micro-organisms (i.e. *Staphylococcus epidermidis* and *Proprionibacterium acnes*) in a homogeneous subgroup of patients with suspected early aseptic failures of THA and TKA.

This is an important limitation in the clinical use of the test, which initially promised to be ideal in simply confirming or excluding any PJI intraoperatively. The sensitivity may be improved by aiming future research at fine-tuning the thresholds of α-defensin in the presence of low-virulent micro-organisms. This may, however, be difficult to achieve since recent reviews failed to establish a more accurate cut-off value. The latter was due to the usage of different techniques among laboratories and a shortage of well-designed studies (Li et al. 2017, Yuan et al. 2017).

It should also be noted that the dichotomic nature of the Synovasure lateral flow test, where the presence or absence of a PJI is claimed, is another limitation. Irrespective of the fact that a solution may be found to decrease the relatively high chance of a false-negative test outcome in case of low-virulent agents, the microbial agents and their resistance patterns would still have to be obtained from prolonged tissue cultures.

For that reason future research should also continue to focus on advances in molecular microbiology and techniques for detecting microbial infections (e.g., susceptibility testing, DNA amplification assays) (Maurer et al. 2017). These techniques may offer increased diagnostic resolution and are not dichotomic by also providing information on the causative pathogen’s identity and resistance pattern. Further improvement on these earlier mentioned microbial detection techniques may eventually bypass the dependency on tissue cultures for adequate antibiotic treatment.  

RS: data collection and writing of the manuscript. JV: study setup and writing of the manuscript. JS: study design, writing of the manuscript. CL: study setup and design, manuscript review.  

*Acta* thanks Inge Skråmm and other anonymous reviewers for help with peer review of this study.
